# Dissolved Organic Phosphorus Removal in Secondary Effluent by Ferrate (VI): Performance and Mechanism

**DOI:** 10.3390/ijerph20042849

**Published:** 2023-02-06

**Authors:** Lei Zheng, Panpan Gao, Yali Song, Hua Wang, Yang Deng

**Affiliations:** 1 School of Civil Engineering and Architecture, Zhejiang University of Science and Technology, Hangzhou 310023, China; 2 Department of Earth and Environmental Studies, Montclair State University, Montclair, NJ 07043, USA; 3 School of Engineering, Hangzhou Normal University, Hangzhou 311121, China

**Keywords:** dissolved organic phosphorus, ferrate (VI), adsorption, secondary effluent, municipal wastewater

## Abstract

Dissolved organic phosphorus (DOP), which is recalcitrant in municipal wastewater treatment, accounts for 26–81% of dissolved total phosphorus in the effluent. More importantly, the majority of DOP could be bioavailable, potentially threatening the aquatic environment through eutrophication. This study aimed to develop a ferrate (VI)-based advanced treatment to effectively destruct and remove DOP from secondary effluent and use deoxyribonucleic acid (DNA) and adenosine-5’-triphosphate (ATP) as DOP model compounds to explore the relevant mechanisms. The results showed that ferrate (VI) treatment could efficiently destruct and remove 75% of the DOP in secondary effluent from an activated sludge-adopted municipal wastewater treatment plant, under normal operating conditions. Moreover, the coexistence of nitrate, ammonia, and alkalinity barely affected the effectiveness, while the presence of phosphate significantly inhibited DOP removal. The mechanistic study revealed that ferrate (VI)-induced particle adsorption was the dominant way to achieve DOP reduction, rather than oxidating DOP to phosphate and forming precipitation afterward. Meanwhile, DOP molecules could be effectively decomposed into smaller ones by ferrate (VI) oxidation. This study clearly demonstrated that ferrate (VI) treatment could achieve a promising DOP removal from secondary effluent for mitigating the risk of eutrophication in receiving water bodies.

## 1. Introduction

Phosphate, the ready form of phosphorus to be utilized by plants and algae, has been the major concern of eutrophication [1]. However, increasing attention has been paid to the underestimated influence of dissolved organic phosphorus (DOP) on surface water eutrophication, water quality, and algal blooms [2,3]. DOP is the dominant form in most freshwater systems and occupies 25–50% of the total phosphorus [4]. In recent years, studies have demonstrated that DOP could be highly bioavailable, for instance, phytoplankton could directly uptake low-molecular-weight DOP for reproduction [5,6,7]. Indeed, DOP and inorganic phosphorus contribute concurrently to the phosphorus source in aqueous ecosystems, and studies have shown that DOP could become a phosphorus source alternative when inorganic phosphorus is absent [8,9]. Therefore, the impacts of DOP on eutrophication in aqueous environments have been ignored, and its influence on water quality impairments and ecosystem health has been underestimated.

As the major discharge source of DOP to surface water, wastewater treatment plants lack effective and efficient processes to address this problem. DOP accounts for approximately 15% of the total phosphorus in municipal wastewater [10]. The conventional wastewater treatment processes mainly target the inorganic phosphorus portion and a very limited amount of DOP gets removed [11]. Although the enhanced biological treatment processes could use the active synthetic metabolism to degrade and utilize DOP for removal [12], microbial metabolism or cell lysis would produce DOP as well [13], leading to the unexpected DOP removal performance. Hence, in the effluent of conventional wastewater treatment plants, DOP remains and dominates 26–81% of the dissolved total phosphorus [14], substantially threatening the health of receiving water bodies. Advanced/tertiary treatments are usually adopted under such circumstances. However, a relevant investigation found that the DOP removal performances using advanced/tertiary treatments were varied, which were significantly correlated with the adopted process, the operation parameters, and the DOP characteristics [15]. The most recent study reported that tertiary treatment units consisting of coagulation, sand filter, and ultraviolet disinfection could significantly remove DOP and achieve a concentration of 0.01 mg/L in the final effluent, but the related chemical, labor, and maintenance costs should be of concern [14]. Therefore, it is urgent to develop an innovative and cost-effective advanced phosphorus removal technique to effectively remove organic phosphorus from the effluent.

Ferrate (VI) is an emerging oxidant with a higher redox potential (2.20 V) than that of ozone (2.08 V) under acidic conditions [16], which has been recognized as an environmental-friendly water treatment agent due to the formation of non-toxic final products (i.e., Fe (III)) and little undesirable disinfection byproducts (DBPs) after ferrate (VI) treatment [17,18,19]. More importantly, it is acknowledged that oxidation/disinfection, coagulation/flocculation, adsorption, and precipitation may happen simultaneously within the ferrate (VI) treatment process [18]. Benefiting from the high valence state of iron, numerous studies have demonstrated that ferrate (VI) was able to oxide various organic compounds and deactivate pathogens and viruses, such as color and odor compounds [19], natural organic matters [20], regulated toxic organic and inorganic species [21,22,23], emerging pollutants [24,25], as well as pathogenic bacteria and viruses [26,27]. Specifically, ferrate (VI) could effectively oxidate parathion, an organophosphate pesticide, into smaller phosphorus-containing molecules and phosphate in aqueous solutions, demonstrating the DOP oxidation potential [28]. In addition, ferrate (VI) reduction could induce stable Fe (III) and inherently cause coagulation and flocculation, facilitating contaminants precipitation and adsorption afterward [29,30,31]. Compared with commonly used coagulants, ferrate (VI)-initiated coagulation could induce faster destabilization of the colloidal particles and further lower turbidity and reduce sludge volume [32]. It was found that ferrate (VI) resultant particles were amorphous iron (III) oxide/oxyhydroxide nanoparticles with a high surface area, which would own great adsorption capability of the tertian type of organic phosphorus [33,34]. In addition, ferrate (VI) treatment demonstrated a promising removal efficiency (up to 97.3%) of inorganic phosphorus during wastewater treatment [35,36,37]. Accordingly, ferrate (VI) treatment has the potential to achieve efficient total phosphorus removal from wastewater by simultaneous oxidation and coagulation.

Herein, this study aimed to investigate organic phosphorus removal from municipal wastewater effluent in detail using ferrate (VI) treatment as an advanced process, explore the impacts of the water matrix on the removal efficiency, and propose the probable mechanisms behind the performance.

## 2. Materials and Methods

### 2.1. Wastewater Samples and Reagents

All the reagents used were at least analytical grade except as noted. Potassium ferrate (K_2_FeO_4_, >96%), sodium bicarbonate (NaHCO_3_), sodium nitrate (NaNO_3_), disodium phosphate (Na_2_HPO_4_), ammonia chloride (NH_4_Cl), deoxyribonucleic acid (DNA) sodium salt, and adenosine-5’-triphosphate (ATP) disodium salt hydrate, were purchased from Sigma-Aldrich (St. Louis, MO, USA). Secondary effluent was collected from a local municipal sewage treatment plant (Verona, NJ, USA). The treatment facility received 9464 m^3^/day of wastewater from residual areas. The secondary effluent was sampled after a secondary clarifier and before disinfection. Once collected, the sample was delivered to Montclair State University’s Innovative Water Treatment and Reuse Laboratory, filtered with 0.45 μm membrane filters (GE Healthcare Whatman™ Nylon Membrane, Waltham, MA, USA), and then stored at 4 °C in a refrigerator until use. Quality parameters of the secondary effluent are included in Table S1. A concentrated ferrate (VI) (200 mg/L Fe (VI)) stock solution was prepared by dissolving appropriate weights of K_2_FeO_4_ in ultrapure water produced with a Milli-Q water purification system (Milli-Q Direct 8). Fe (VI) in the ferrate (VI) stock solution was confirmed with the ABTS method [38]. Organic phosphorus model compound-containing solutions were prepared using the dissolution of appropriate weights of DNA and ATP in ultrapure water to ensure the initial DOP at 500 µg/L, respectively. The initial DOP concentration was confirmed using an inductively coupled plasma-mass spectroscopy (ICP-MS Thermo X-Series II, XO 472). The solutions were prepared immediately before the treatment tests.

### 2.2. Ferrate (VI) Removal of DOP in Secondary Effluent

Laboratory-scale batch tests were carried out in 500 mL glass beakers containing 400 mL of well-mixed secondary effluent on a four-paddle programmable jar tester (Phipps and Bird—7790-950) at room temperature (20 ± 1 °C) and under atmospheric pressure. If needed, the secondary effluent pH was adjusted to a designated level with 0.1 M sulfuric acid or sodium hydroxide. The treatment was initiated once an appropriate volume of ferrate (VI) stock solution was dosed. Within the first minute, the solution was rapidly mixed at a velocity gradient of 241 s^−1^ to completely disperse the added iron. During the following 59 min, the solution was gently stirred at a velocity gradient of 21 s^−1^ for the growth of flocs. During the treatment, pH was monitored, but not controlled or intendedly buffered. Following the slow mixing, the treated wastewater was filtered using 0.45 μm membrane filters (GE Healthcare Whatman™ Nylon Membrane) for removing particulate matter, and the filtrates were collected for further analysis. Control tests were carried out with the identical experimental procedure, except that ferrate (VI) was not dosed.

In the tests to evaluate the effect of ferrate (VI) dose, the ferrate (VI) dose was varied from 0.0 to 9.0 mg/L. In the experiments to assess the effect of initial pH, the ferrate (VI) dose was fixed at 5.0 mg/L Fe, while the initial pH was varied from 5.5 to 8.0. For the experiments to evaluate the effects of wastewater matrix constituents, alkalinity (250–500 mg/L as CaCO_3_), nitrate (NO_3_¯, 2.6–30.0 mg/L NO_3_¯-N), ammonia (NH_3_, 10–60 mg/L NH_3_-N), and disodium phosphate (Na_2_HPO_4_, 2.08–5.22 mg/L as P) were dosed to achieve their respective designated concentrations, and the treatment tests were carried out at 5.0 mg/L Fe (VI) and pH 6.5.

### 2.3. Ferrate (VI) Treatment of DOP Model Compounds

*In-situ* ferrate (VI) treatment tests for the removal of individual DOP model compounds were carried out with a procedure similar to the treatment tests for secondary effluent except that: (1) a DOP solution, rather than secondary effluent, was treated; and (2) pH was manually controlled at a designated pH using 0.1 M sulfuric acid and sodium hydroxide. On the other hand, *ex-situ* ferrate (VI) treatment tests in the DOP solution were performed using a similar *in-situ* treatment procedure. The only modification is that ferrate (VI) was dosed first and then mixed for 1 h before the DOP model compound was introduced. *In-situ* and *ex-situ* treatment tests would evaluate the combined effect of ferrate (VI) oxidation and iron (hdyr)oxides adsorption and the effect of iron (hdyr)oxides adsorption only, respectively. After the *in-situ* or *ex-situ* treatment, samples were filtered through 0.45 μm membrane filters (GE Healthcare Whatman™ Nylon Membrane) to remove particulate matter, and the filtrate was collected for further analyses. In the tests for determination of the size distributions of the ferrate (VI) resultant particles, the samples, after treatment, comprising water and iron (hdyr)oxides, were sequentially filtered through 0.45 μm microfiltration (GE Healthcare Whatman™ Nylon Membrane), 0.1 μm microfiltration (Thermo Scientific, cellulose acetate (CA), Waltham, MA, USA), and 30 kDa ultrafiltration (UF) membranes (EMD Millipore, regenerated cellulose, Burlington, MA, USA). Approximately 50 mL of filtrate after each filtration was collected for analysis. Particles filtered with 0.45 μm microfiltration were operationally defined as large-sized particles. Solids passing 0.45 μm microfiltration filters but filtered with 0.1 μm microfiltration were colloidal particles. Particles passing 0.1 μm microfiltration filters but filtered with a 30 kDa UF membrane were nano-sized particles. The chemicals passing 30 KDa MF filters were regarded as soluble substances. A similar fractionation method was applied elsewhere to study the size of iron particles in ferrate (VI) decomposition in natural waters [39].

### 2.4. Sample Analyses

Ferrate (VI) was spectrophotometrically measured using the ABTS method [38]. Solution pH was measured with a pH meter (Thermo Scientific Orion 5-Star Plus). Various secondary effluent parameters were measured after filtration with 0.45-μm syringe membrane filters (Thermo Scientific, cellulose acetate (CA), 30 mm diameter). Effluent organic matter (EfOM) was quantified using dissolved organic carbon (DOC), which was measured with a total organic carbon (TOC) analyzer (TOC-LCPH, Shimadzu Corp., Kyoto, Japan). Measurements of alkalinity, NO_3_¯-N, and NH_3_-N followed the U.S. Environmental Protection Agency (EPA) approved Standard Methods 2320B, 1685, and 4500F, respectively. Total phosphate was determined with an inductively coupled plasma-mass spectroscopy (ICP-MS Thermo X-Series II, XO 472). Acid hydrolyzable phosphorus was measured with the HACH TNT reagent sets to indicate inorganic phosphorus in the water. DOP was the difference between total phosphorus and inorganic phosphorus. The morphology of the retained particles was examined with a transmission electron microscope (TEM, Hitachi H-7500, Tokyo, Japan) and a scanning electron microscope (SEM, Hitachi S-3400N). The daughter products of model DOPs were identified using a liquid chromatography–mass spectrometry system (LC-MS 2020, Shimadzu Corp., Kyoto, Japan), which was equipped with a Supelcosil LC-18 column (25 cm × 4.6 mm, 5 μm). Mobile phase A consisted of 100% acetonitrile, while mobile phase B consisted of 7.5 mM of ammonium acetate and adjusted to pH = 7.5 with 0.1 mol/L acetate acid and ammonium hydroxide. The flow rate was controlled at 0.5 mL/min with a retention time of 10 min in total. The ratio of mobile phases A and B was fixed at 35% and 65% throughout the analysis. The injection volume was 20 μL, and the peaks were detected at 254 nm.

All the experiments were run, at a minimum, in triplicates. All the analytical results reported represent the mean of the replicate samples. Error bars in the figures indicate one standard deviation of these measurements.

## 3. Results

### 3.1. Dissolved Organic Phosphorus Removal from Secondary Effluent

#### 3.1.1. Effect of Ferrate (VI) Dose

**Figure 1:**
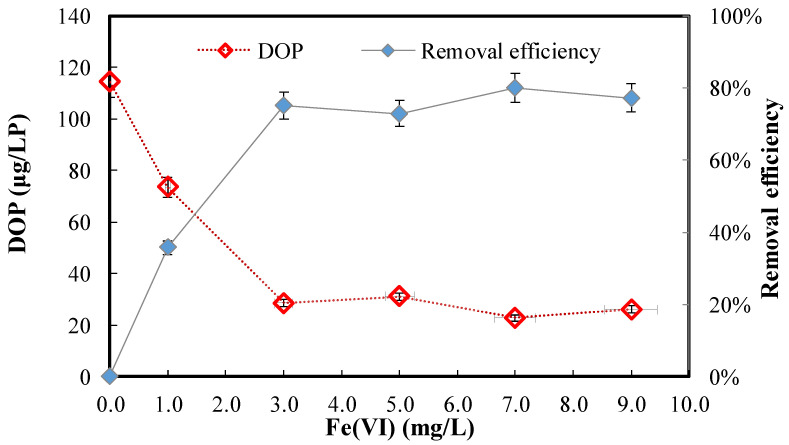
Residual DOP and DOP removal efficiency after ferrate (VI) treatment of secondary effluent at different Fe (VI) doses. (DOP = 114 μg/L as P; Fe (VI) = 0.0–9.0 mg/L).

The residual dissolved organic phosphorus (DOP) and DOP removal efficiency after ferrate (VI) treatment of the secondary effluent at different Fe (VI) doses are shown in Figure 1 (DOP = 114 μg/L as P; Fe (VI) = 0.0–9.0 mg/L). As shown, as the Fe (VI) dose was increased from 0.0 to 3.0 mg/L, the residual DOP sharply declined from 114 to 29 µg/L, while the DOP removal efficiency dramatically increased from 0% to 75%. As the Fe (VI) dose was further increased to 9.0 mg/L, the residual DOP almost stabilized at 29–31 µg/L with a corresponding DOP removal efficiency ranging within 75–80%. Since 3.0 mg/L could not ensure the initiation of coagulation [40], the Fe (VI) dose was selected at 5.0 mg/L for the following experiments. These findings clearly demonstrate that ferrate (VI) could effectively alleviate the DOP in secondary effluent.

#### 3.1.2. Effect of pH

**Figure 2:**
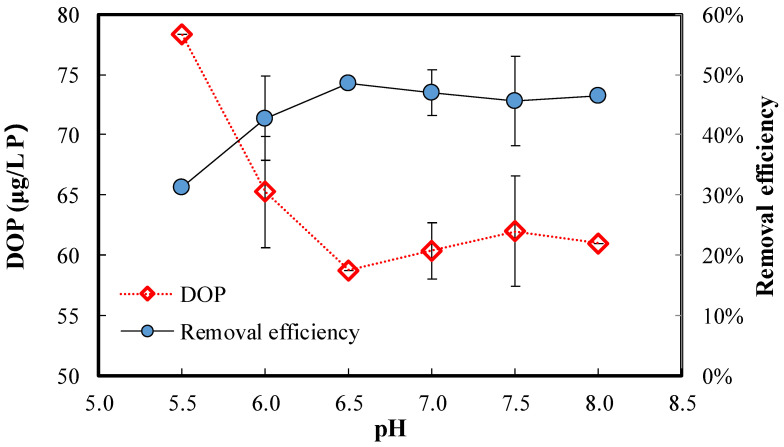
Residual DOP and DOP removal efficiency after ferrate (VI) treatment of secondary effluent at different initial pH. (DOP = 114 μg/L as P; Fe (VI) = 5.0 mg/L; and initial pH = 5.5–8.0).

The residual DOP and DOP removal efficiency after ferrate (VI) treatment of secondary effluent at different initial pH are shown in Figure 2 (DOP = 114 μg/L as P; Fe (VI) = 5.0 mg/L; and initial pH = 5.5–8.0). The residual DOP dramatically decreased from 78 μg/L at pH 5.5 to 59 μg/L at pH 6.5, while the removal efficiency was increased from 31% to 49%. As pH further increased to 8.0, the residual DOP was not obviously altered. These observations evidently show that pH disfavors the DOP removal with the pH decreased to an acidic solution condition. However, the effect of pH on the DOP removal was insignificant at a weakly acidic to weakly alkaline condition (pH 6.5–8.0). Therefore, the solution pH was selected at 6.5 for the following experiments.

#### 3.1.3. Effect of Co-Existing Ions

**Figure 3:**
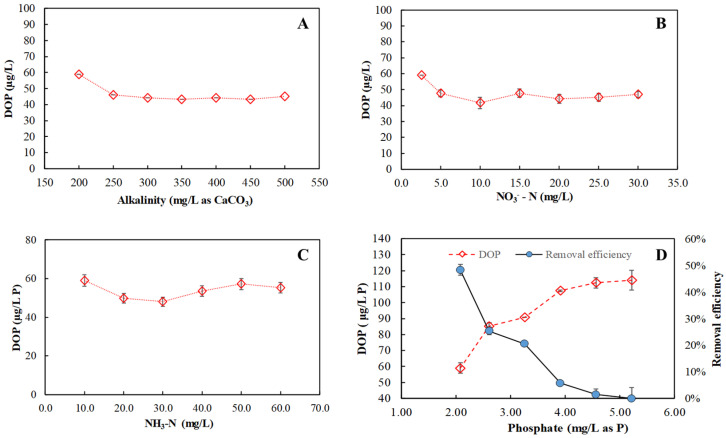
Effects of different wastewater matrix constituents on residual DOP during ferrate (VI) treatment of secondary effluent. (Fe (VI) dose = 5.0 mg/L; initial DOP = 114 µg/L P; and initial pH = 6.5) (**A**) alkalinity: 200–500 mg/L as CaCO_3_; (**B**) NO_3_¯-N: 2.6–30.0 mg/L; (**C**) NH_3_-N: 10.6–60 mg/L; and (**D**) phosphate: 2.08–5.22 mg/L as P.

The effects of common wastewater matrix constituents, including alkalinity, NO_3_¯, NH_4_^+^, and PO_4_^3−^, were subsequently investigated, as shown in Figure 3. As seen in Figure 3A, residual DOP slightly declined from 59 µg/L (48% removal) at an alkalinity of 200 mg/L CaCO_3_ to 47 µg/L (59% removal) at an alkalinity of 250 mg/L CaCO_3_, and then narrowly varied between 44 and 47 µg/L, corresponding to the removal efficiency within 59%–69%, over an alkalinity range of 250–500 mg/L CaCO_3_, indicating that alkalinity did not greatly affect the DOP removal. The effect of NO_3_¯ on the residual DOP is illustrated in Figure 3B. When NO_3_¯-N was increased from 2.6 to 5.0 mg/L, the residual DOP dropped from 59 to 48 µg/L, corresponding to the increase in the DOP removal efficiency from 48% to 58%. At 5.0–30.0 mg/L NO_3_¯-N, the residual DOP ranged within 42–48 µg/L, also indicating that nitrate had a minor influence on ferrate (VI) removal of organic phosphorus in secondary effluent. The effect of NH_3_ on the residual DOP is presented in Figure 3C. Over 10.6–60.0 mg/L NH_3_-N, the residual DOP varied slightly within 48–59 µg/L, corresponding to the overall DOP removal of 48–58%, suggesting that ammonia had a limited influence on ferrate (VI) removal of DOP in secondary effluent. Overall, these observations indicated that the impacts of alkalinity, nitrate, and ammonia on the organic phosphorus removal during ferrate (VI) treatment of secondary effluent were insignificant.

In contrast, a significant inhibiting effect of inorganic orthophosphate on ferrate (VI) removal of DOP was observed, as shown in Figure 3D. As seen, as phosphate increased from the original 2.08 to 5.22 mg/L as P (i.e., 16.00 mg/L PO_4_^3-^), the residual DOP increased from 59 to 112 µg/L, with the corresponding removal efficiency dramatically declining from 48% to 1%, indicating that the DOP removal was suppressed with the increasing phosphate concentration.

### 3.2. Dissolved Organic Phosphorus Removal Mechanism Exploration

#### 3.2.1. Model Dissolved Organic Phosphorus Removal

**Figure 4:**
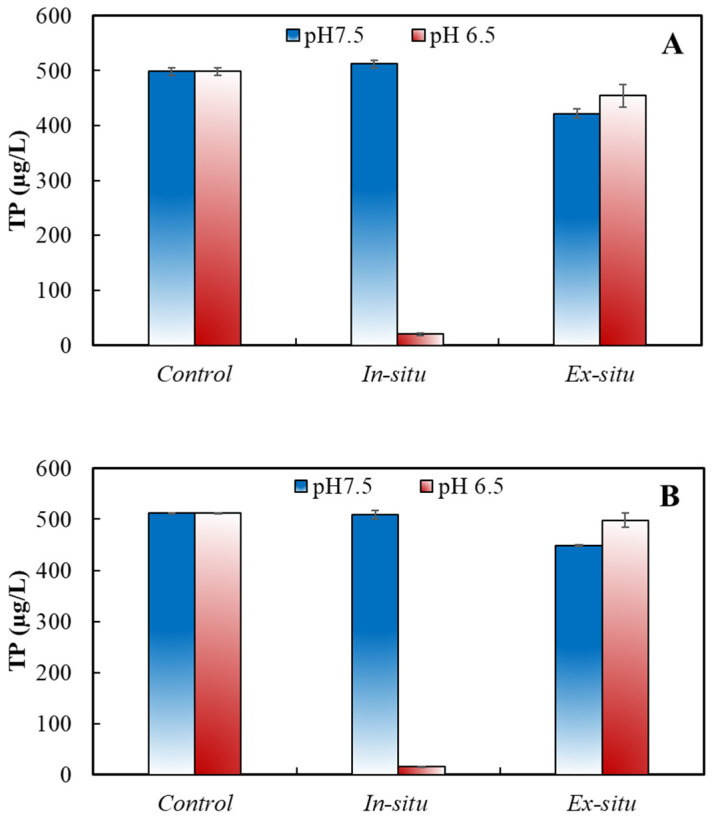
Ferrate (VI) removal of different model DOP compounds at different conditions: (**A**) DNA; (**B**) ATP. (TP = 500 μg/L as P; Fe (VI) = 5.0 mg/L; pH = 6.5 or 7.5; Control: no ferrate (VI) addition; *in-situ* treatment: direct ferrate (VI) addition to DOP-containing solution; and *ex-situ* treatment: ferrate (VI) decay followed by DOP addition).

The residual DOP concentrations after ferrate (VI) treatment of DNA and ATP are shown in Figure 4 (Fe (VI) = 5.0 mg/L; P = 500 µg/L). For each model compound, *in-situ* and *ex-situ* ferrate (VI) treatments were performed at pH 6.5 or 7.5. The *in-situ* treatment meant that ferrate (VI) was dosed to the P-containing water, in which ferrate (VI)-driven oxidation and adsorption might both contribute to the DOP removal. On the other hand, in an *ex-situ* treatment, ferrate (VI) was dosed with distilled water and then depleted before a specific DOP model was introduced, which reflected the DOP adsorption effect only. Of note, partial or whole DOP model molecules may be subject to a change in chemical structure due to chemical oxidation during an *in-situ* treatment, while the structure of the DOP molecules remained intact over the *ex-situ* treatment because of the lack of chemical degradation reactions.

As shown in Figure 4, the residual P concentrations of the two DOP model compounds all slightly declined (<20%) in comparison to their respective initial levels at either pH during the *ex-situ* ferrate (VI) treatment. The finding evidently demonstrated that there was a poor adsorption of ferrate (VI) resultant particles for the model compounds. Therefore, direct adsorption due to the formation of iron (hydr)oxide did not serve as a principal mechanism for mitigation of these model compounds.

During the *in-situ* treatment, all model compounds were very little removed at pH 7.5, indicating that the joint utilization of chemical oxidation and adsorption could not effectively remove these compounds at the studied conditions. However, the residual P significantly declined at pH 6.5 after the *in-situ* treatment. The residual P in DNA and ATP decreased from the initial 500 µg/L to 15 and 2 µg/L, respectively. Based on the aforementioned results, we can conclude that the effective removals of DNA and ATP are primarily ascribed to the co-occurrence of ferrate (VI)-driven chemical oxidation and adsorption at a weakly acidic condition.

#### 3.2.2. Size Fractions of P and Fe after Ferrate (VI) Treatment

In order to further understand the size fractions of P and Fe after the *in-situ* treatment, the treated samples were subject to sequential filtration with 0.45 µm microfiltration, 0.1 µm microfiltration, and 300 kDa ultrafiltration, respectively. Concentrations of residual P and Fe in the filtrate after each filtration were measured. Total P and Fe in the ferrate (VI) treated samples were also analyzed after sample digestion. In this study, particulate matter with sizes of >0.45 µm, 0.45–0.1 µm, and 0.1 µm–300 kDa were categorized into large-sized, colloidal, and nano-sized particles, respectively. P and Fe measured in the filtrate after <300 kDa ultrafiltration derived from soluble substances. The size fractionation methods were successfully adopted for investigation of the size distribution patterns of these ferrate (VI) resultant particles, which were produced after ferrate (VI) treatment of natural water or sewage-polluted source water in previous studies [41].

**Figure 5:**
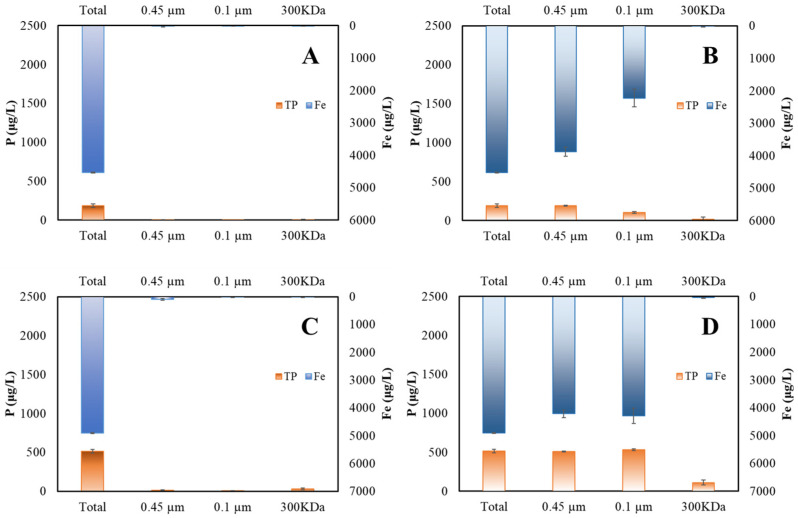
Size distribution of P and Fe after *in-situ* ferrate (VI) treatment of different DOP model compounds: (**A**) DNA *in-situ* at pH 6.5; (**B**) DNA *in-situ* at pH 7.5; (**C**) ATP *in-situ* at pH 6.5; and (**D**) ATP *in-situ* at pH 7.5. (initial DOP = 1000 μg/L as P; Fe (VI) = 5.0 mg/L).

The size distributions of P and Fe after ferrate (VI) treatment of DNA and ATP at pH 7.5 are shown in Figure 5B,D. At pH 7.5, P and Fe shared similar size distribution patterns. Two important findings were observed during the experiments. First, for any specific model compound, soluble P and Fe were insignificant, suggesting that P was not indeed dissolved in the treated water. Second, the majority of P and Fe were present in the nano-sized particles, implying that most P adsorbed to or was incorporated into ferrate (VI) resultant particles. Because partial or whole parent compounds may be degraded by ferrate (VI) oxidation, we cannot conclude whether the P associated with the nano-sized particles originated from the parent compounds, daughter compounds, or even inorganic phosphate, which might be produced from ferrate (VI) oxidation of DOP molecules, based on the above observations.

The size distributions of P and Fe after ferrate (VI) treatment at pH 6.5 are shown in Figure 5A,C. Very different size distribution patterns of Fe and P were observed. The vast majority of P and Fe in the DNA and ATP treatment groups existed in the large-sized particles of >0.45 µm. Based on these findings, we could acquire important information in the three aspects. First, the very low concentrations of the soluble P indicated that residual P was not truly dissolved after the treatment. Second, the similar size distributions of P and Fe implied that P was associated with ferrate (VI) resultant particles with different sizes. Third, the sizes of these particles produced at lower pH were greater than those at higher pH, which favored the downstream filtration that could remove the P associated with the particles. This finding was likely related to the performance of iron-driven coagulation.

From this set of experiments, we noticed that the DNA used contained very low fractions of DOP in terms of mass. To achieve 500 µg/L DOP for the above experiments, too much DNA needed to be dosed into the water, which had a much greater DOC than the realistic DOC in secondary effluent. That is, DNA had a very high mass ratio of C to P. Given that the concentrations of DNA should be trivial in a real secondary effluent, DNA was not investigated in the following experiments.

#### 3.2.3. Surface Element Analyses of Ferrate (VI) Resultant Particles

**Figure 6:**
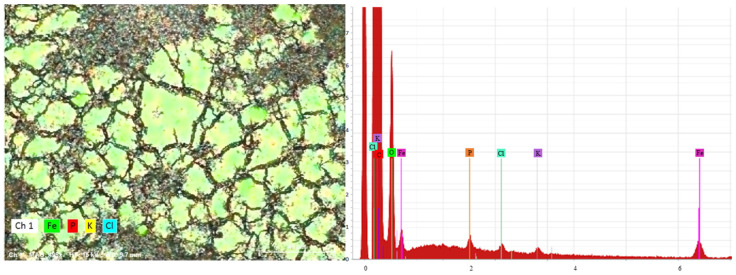
Surface element analysis of ferrate (VI) resultant particles after ferrate (VI) treatment of ATP in distilled water. (P = 500 µg/L; Fe (VI) = 5.0 mg/L; pH = 6.5).

Energy dispersive spectrum (EDS) analyses were performed for the particles produced from the *in-situ* ferrate (VI) treatment of ATP at pH 6.5, as shown in Figure 6. The results showed that abundant Fe and P coexisted in these particles, which was in agreement with the above observation that both P and Fe shared similar distribution patterns.

#### 3.2.4. Identification of Degradation Products in Treated Water

**Figure 7:**
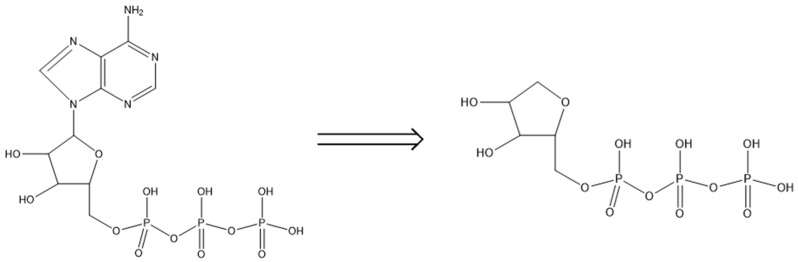
Proposed degradation pathway of ATP by ferrate (VI) oxidation. (P = 500 µg/L; Fe (VI) = 5.0 mg/L; and pH = 6.5).

In order to understand the reactions of ferrate (VI) and DOP model compounds, the degradation products of ATP after ferrate (VI) oxidation were identified using LC/MS. *In-situ* ferrate (VI) treatment tests were performed at pH 6.5. LC/MS data of the parent and major daughter compounds after ferrate (VI) treatment of ATP are shown in Figure S1, and the proposed degradation pathway is shown in Figure 7. After treatment, a major daughter compound with a P-containing chain was identified. Of interest, the daughter compound of ATP was a fragment after the breakdown of the C–N bond between two ring structures in the parent compound. Ferrate (VI) reactivity toward different organic molecules was extensively investigated [42,43]. The ferrate (VI)-induced degradation may be involved with electron transfer, hydrogen atom transfer, hydride ion, or covalently bonded ferrate intermediates depending upon the nature of the compound. Particularly, similar to ozone, ferrate (V) preferentially attacks electron-rich function groups on an organic molecule [43]. The C–N bonds of two ring structures on the parent compounds were attacked due to the electron-rich property. The cleavage occurred due to the low bond energy and easy activation through the resonance from the two ring structures. Cleavage of the C–N bond by ferrate (VI) oxidation was also previously reported elsewhere [44,45].

## 4. Discussion

### 4.1. Mechanisms for DOP Removal with Ferrate (VI)

Ferrate (VI) oxidation may entirely or partially degrade DOP compounds into daughter P-containing compounds or into inorganic P. Following ferrate (VI) treatment, the P originally present in these DOP model compounds would have three possible fates, including (1) parent compounds in water or on the ferrate (VI) resultant particles; (2) daughter DOP compounds in water or on the ferrate (VI) resultant particles; and (3) inorganic phosphorus in water or associated with the ferrate (VI) resultant particles. Further analyses of the fate of P after ferrate (VI) treatment is of importance to understand the reaction mechanisms for DOP in secondary effluent. The detailed discussion is further analyzed on ferrate (VI) removal of the selected DOP model compounds.

The results from the *ex-situ* ferrate (VI) treatment showed a poor removal of these DOP model compounds through adsorption only with the ferrate (VI) resultant particles. Therefore, it is unlikely that these parent compounds could be mostly captured by the produced iron (hydr)oxides. Meanwhile, we did not find these parent compounds in water after ferrate (VI) treatment. Therefore, the selected DOP compounds were almost all degraded by ferrate (VI) oxidation.

On the other hand, inorganic P (e.g., orthophosphate and polyphosphate) was undetectable in the aqueous phase after ferrate (VI) treatment. It is most unlikely that polyphosphate, if truly produced from the DOP degradation, could adsorb to iron (hydr)oxide because polyphosphate is poorly removed by adsorption with iron (hydr)oxide [32]. The extraction method was used to examine whether any orthophosphate was adsorbed to the ferrate (VI) resultant particles. The results showed the absence of orthophosphate in the iron sludge. The aforementioned findings suggested that ferrate (VI) could not sufficiently degrade these DOP model compounds into inorganic phosphorus. It is well known that ferrate (VI) preferentially transforms dissolved organic matter, rather than completely oxidizing it into inorganic species [20,43]. This was evidenced by the fact that ferrate(VI) oxidation could readily alleviate chemical oxidation demand (COD) but poorly abate DOC when it was used for the degradation of NOM in natural water sources [20]. This finding was primarily ascribed to the nature of ferrate (VI). Although it has a high redox potential of up to 2.20 V, ferrate (VI) oxidation is highly selective. The oxidative anion tends to react with electron-rich moieties, but very slowly reacts with electron-poor moieties. Meanwhile, ferrate (VI) is subject to its self-decomposition in water, which can make ferrate (VI) gradually decay until ferrate (VI) depletion.

Based on the mass balance analysis, only daughter P-containing compounds existed in the treatment systems. The degradation of the parent compounds could be validated with the identification of decomposition products in solutions after the treatment using the mass spectrometry technique. The results in the ferrate (VI) treatment of the selected DOP model compounds clearly indicated that most of the P was associated with the finally produced iron (hydr)oxides. Therefore, the P was plausibly present in the degradation products of the DOP model compounds after ferrate (VI) treatment. Overall, the major plausible reaction mechanisms for ferrate (VI) removal of the selected DOP compounds are as follows. Ferrate (VI) first attacks certain functional groups on the DOP parent compounds for the production of various P-containing compounds. The chemical oxidation most likely occurs in electron-rich moieties. With the nature of different degradation products, some sorb to the produced iron (hydr)oxides, while the others remain in the water in a soluble state. Adsorption of P-containing organic compounds to various iron oxides has been documented elsewhere [34,46,47].

It should be noted that the aforementioned reaction pathways for ferrate (VI) oxidation and ensuing adsorption for the removal of DOP are proposed based on the experimental evidence with selected DOP model compounds. Organic phosphorus in EfOM may have been contributed from many other individual organic compounds in the secondary effluent. Further studies are needed for an in-depth understanding of ferrate (VI) reactions with other DOP species in EfOM.

### 4.2. Operation Factors and Coexisting Wastewater Matrix Constituents

Ferrate (VI) removal of DOP, similar to ferrate (VI) alleviation of phosphate in water, can be greatly influenced by the ferrate (VI) dose and solution pH. Better DOP removal was achieved at a higher ferrate (VI) dose during the treatment of secondary effluent because more Fe (VI) was available for the degradation of DOP compounds and more iron (hydr)oxide particles were produced for adsorption of the DOP degradation products. On the other hand, a lower pH of 5.5–6.5 favored DOP removal likely due to the greater ferrate (VI) reactivity. Ferrate (VI) reactivity is acutely increased with a pH decrease [16]. The extremely strong Fe (VI) at such an acidic condition may greatly degrade DOP into inorganic phosphate before the removal of P through adsorption. However, in realistic municipal wastewater treatment, solution pH mostly varies within a nearly neutral pH range (6.5–8.5). In the treatment tests with real secondary effluent, a very slight difference was observed for the DOP removals over pH 6.5–8.0. However, in the ferrate (VI) treatment tests for model DOP compounds, better removal was observed at pH 6.5 than at pH 7.5. As discussed above, the difference was caused due to the different size distribution patterns of ferrate (VI) resultant particles produced at different pH. Generally, more large-sized particles were produced at pH 6.5, so that these particles to which DOP compounds adsorb were readily removed in the downstream filtration, thereby having a lower residual DOP.

Different from many other wastewater matrix constituents without a marked effect on ferrate (VI) removal of DOP, phosphate exhibited a significant inhibiting effect. Adsorption of phosphate to ferrate (VI) resultant particles was the dominant mechanism for ferrate (VI) removal of reactive phosphate in secondary effluent. More phosphate was expected to be captured by the ferrate (VI)-induced particles when more phosphate was present in the secondary effluent. The ferrate (VI) treatment tests for the removal of model DOP compounds also revealed that the adsorption of P-containing degradation compounds to ferrate (VI) resultant particles played an essential role in the DOP alleviation. Therefore, there was a competition between phosphate and DOP compounds for active adsorption sites on iron (hydr)oxides. More adsorption sites would be occupied at a higher initial phosphate concentration, so that fewer DOP compounds were adsorbed. Additionally, phosphate had a greater affinity with various iron oxides than many organic phosphorus compounds [46].

### 4.3. Implication to the Wastewater Industry

The findings of this work have unique implications for the wastewater industry in the following aspects:

(1) This study finds a new pathway to prevent recalcitrant wastewater-derived DOP from entering natural receiving water bodies. The addition of common coagulants, such as ferric chloride and aluminum sulfate, has been used as a widely accepted method for the elimination of phosphate from biologically treated municipal wastewater due to their effectiveness and low costs. Although coagulation can well remove reactive phosphate, it has proven ineffective for the mitigation of DOP. Encouraging results from this study show that ferrate (VI) is a promising treatment agent for capturing DOP in secondary effluent. This is of significance, particularly for the natural water bodies that are highly environmentally sensitive to nutrient loadings (e.g., the Chesapeake Bay in the United States).
(2) Ferrate (VI) removal of DOP can be significantly influenced by the ferrate (VI) dose, water pH, and the presence of phosphate. The first two are operating factors, while the last one represents a wastewater matrix constituent. For different secondary effluents, the specific optimal ferrate (VI) dose and pH need to be determined. It should be noted that the original secondary effluent pH may not be the optimal level. If an additional pH adjustment is required before and after the treatment, the costs are increased due to the use of acid/base as well as additional pH adjustment equipment and pipelines. Accordingly, the system design, operation, and maintenance would become more complex. If this is the case, what ferrate (VI) dose and pH are realistically adopted depends on the comparison at different operational conditions in terms of treatment efficiencies and treatment expenses.
(3) This study reveals that DOP adsorption to ferrate (VI) resultants plays a vital role in the ferrate (VI) removal of wastewater DOP. The controlling of particle sizes during the treatment operation is essential to selection the of appropriate downstream liquid–solid separation techniques, because different liquid–solid separation methods are effective for different particle size ranges. Generally, larger particles are more readily and less costly separated. Therefore, the size growth of particles during ferrate (VI) treatment through appropriate engineering designs and operation control (e.g., control of pH and chemical mixing gradients) deserves a further investigation in future.

## 5. Conclusions

In this study, the treatment performance of ferrate (VI) removal of DOP in secondary effluent was evaluated. The effects of two operating factors (i.e., ferrate (VI) dose and water pH) and four coexisting wastewater matrix constituents (i.e., alkalinity, nitrate, ammonia, and phosphate) were particularly evaluated. In order to elucidate the underlying reaction mechanisms, ferrate (VI) reactions with two DOP model compounds were investigated. The major conclusions from this study are summarized as follows:

(1) Ferrate (VI) treatment is a technically effective method for the mitigation of organic phosphorus in biologically treated municipal wastewater. The treatment efficiency is influenced by the ferrate (VI) dose and pH.
(2) Among different wastewater matrix constituents, alkalinity, nitrate, and ammonia have very limited influence on ferrate (VI) removal of DOP. In contrast, inorganic phosphate can suppress the DOP removal. The inhibiting effect is ascribed to the competitive adsorption of phosphate with OP compounds for active adsorption sites on the ferrate (VI) resultant particles.
(3) Based on the studies with DOP model compounds, ferrate (VI) oxidation and ensuing adsorption with ferrate (VI) resultant particles are the major DOP removal mechanisms. Specifically, ferrate (VI) firstly degrades these parent DOP compounds into daughter P-containing compounds, followed by the adsorption of these daughter compounds by the resultant iron (hdyr)oxide particles. Of note, inorganic phosphate is not observed after ferrate (VI) treatment of DOP model compounds, suggesting that ferrate (VI) insufficiently degrades DOP into inorganic P. However, the conclusions are made only based on the selected model compounds. Given that DOP derives from various EfOM molecules, novel experimental approaches need to be developed for the exploration of the mechanisms for ferrate (VI) removal of aggregate DOP in secondary effluent.

## Data Availability

The data in this study are available within the article and Supplementary Files.

## Supplementary Materials

The following supporting information can be downloaded at: https://www.mdpi.com/article/10.3390/ijerph20042849/s1, Figure S1: LC-MS information of ATP (A: LC detection of pure ATP; B: MS of ATP molecule; C: MS of daughter product); Table S1: Water quality of sampled secondary effluent.
