# Supplementary material for: Dissolved Organic Phosphorus Removal in Secondary Effluent by Ferrate (VI): Performance and Mechanism
Source: Int J Environ Res Public Health. 2023 Feb 6;20(4):2849. doi: 10.3390/ijerph20042849 (PMC9956993; doi:10.3390/ijerph20042849)
Supplement: Supplementary file 1 [file ijerph-20-02849-s001.zip › ijerph-2180057-supplementary.pdf]

Supplementary Materials for

Dissolved organic phosphorus removal in secondary effluent by ferrate (VI): performance and mechanism

Lei Zheng[1,2], Panpan Gao[3], Yali Song[1], Hua Wang[1], Yang Deng[2,*]

[1] School of Civil Engineering and Architecture, Zhejiang University of Science and Technology, Hangzhou 310023, China

[2] Department of Earth and Environmental Studies, Montclair State University, Montclair, NJ 07043, USA

[3] School of Engineering, Hangzhou Normal University, Hangzhou 311121, China

*Corresponding author.

Prof. Yang Deng

CELS Room 220, Montclair State University, 1 Normal Ave, Montclair, NJ 07043, USA.

Email: dengy@mail.montclair.edu

Table S1 Water quality of sampled secondary effluent

Parameters	Value
pH	7.48
UV_{254} absorbance	0.161 cm^{-1}
Turbidity	2.33 NTU
Dissolved organic carbon (DOC)	40.0 mg/L
Alkalinity	200 mg/L as $CaCO_3$
Dissolved total phosphorus (DTP)	2.12 mg/L as P
Dissolved inorganic phosphorus (DIP)	2.08 mg/L as P
Total organic phosphorus (TOP)	42.0 μg/L as P
Nitrate-nitrogen (NO_3^--N)	2.60 mg/L
Ammonia-nitrogen (NH_3-N)	10.4 mg/L

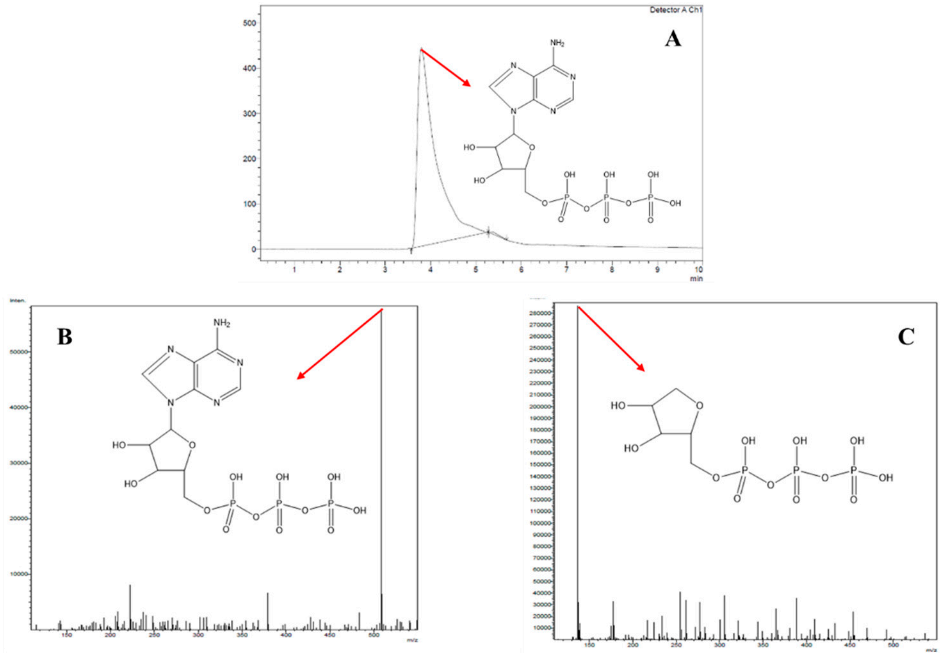

Figure S1 LC-MS information of ATP (A: LC detection of pure ATP; B: MS of ATP molecule; C: MS of daughter product)
